# Combining Grape Byproducts to Maximise Biological Activity of Polyphenols in Chickens

**DOI:** 10.3390/ani11113111

**Published:** 2021-10-30

**Authors:** Carlos Romero, Maria Nardoia, Agustín Brenes, Ignacio Arija, Agustín Viveros, Susana Chamorro

**Affiliations:** 1 Facultad de Ciencias y Artes, Universidad Católica Santa Teresa de Jesús de Ávila (UCAV), Calle Canteros s/n, 05005 Ávila, Spain; carlos.romero@ucavila.es; 2 Department of Agricultural, Environmental and Food Sciences, University of Molise, 86100 Campobasso, Italy; maria.nardoia@studenti.unimol.it; 3 Instituto de Ciencia y Tecnología de Alimentos y Nutrición (ICTAN-CSIC), José Antonio Novais, 10, Ciudad Universitaria, 28040 Madrid, Spain; agustin.brenes@hotmail.com; 4 Department of Animal Production, Faculty of Veterinary Medicine, Complutense University of Madrid, 28040 Madrid, Spain; arijai@ucm.es (I.A.); viverosa@ucm.es (A.V.); 5 Department of Genetics, Physiology and Microbiology (Animal Physiology Unit), Faculty of Biology, Complutense University of Madrid, 28040 Madrid, Spain

**Keywords:** grape polyphenols, grape seeds, grape skins, α-tocopherol, antioxidant activity, chickens

## Abstract

**Simple Summary:**

Grape byproducts (grape pomace, seeds and skins) contain a wide range of phenolic compounds with antioxidant properties and thus can become functional ingredients in animal feeds. The dietary inclusion of grape pomace in chicken diets has been shown to increase plasma and meat α-tocopherol content and to mitigate meat lipid oxidation. However, the separate inclusion of the main components of grape pomace (grape seeds and skins) in the diet of chickens has been less studied. In the present research work, the dietary inclusion of grape byproducts did not compromise the growth of chickens nor did it affect ileal protein digestibility. Concerning plasma and meat α-tocopherol concentrations and meat lipid oxidation, the dietary combination of grape seeds and skins provided better results than the individual inclusion of these grape byproducts. Actually, combinations with a proportion of at least 50% of grape skins enabled optimal results of meat oxidative stability.

**Abstract:**

Grape seeds (GS) and grape skins (GK) are natural sources of polyphenols with antioxidant capacity. An experiment was conducted to investigate in chickens the effect of including GS and GK (40 g/kg), individually or combined in different proportions (20 g/kg GS–20 g/kg GK; 30 g/kg GS–10 g/kg GK; 10 g/kg GS–30 g/kg GK), in a corn-soybean diet on growth performance, ileal and excreta contents of total extractable polyphenols (TEP) and tannins, ileal digestibility of protein, plasma and meat α-tocopherol concentration and lipid oxidation (assessed by measuring the thiobarbituric acid reactive substances, TBARS) of stored thigh meat. Neither growth performance parameters nor ileal digestibility of protein were affected by dietary treatments. As compared with control birds, chickens fed the grape byproduct diets showed higher ileal (*p* < 0.001) and excreta (*p* < 0.001) TEP and tannins contents. Dietary inclusion of grape byproducts increased α-tocopherol concentration both in plasma (*p* < 0.001) and in thigh meat (*p* < 0.01 at 1 d; *p* < 0.001 at 7 d), as compared with the control group. The highest plasma α-tocopherol concentrations were reached with the 30 g/kg GS–10 g/kg GK and 20 g/kg GS–20 g/kg GK combinations. On day 1 of meat storage, no differences on meat α-tocopherol concentration were found among the grape byproducts treatments but on day 7 of storage, the 20 g/kg GS–20 g/kg GK and 10 g/kg GS–30 g/kg GK combinations led to the highest α-tocopherol concentrations in chicken thigh meat. After seven days of refrigerated storage of meat, the TBARS value was lower in chickens fed the grape byproducts diets than in control birds (1.27 vs. 2.49 mg MDA/kg, *p* < 0.001). Moreover, among the different grape byproduct treatments, the lowest MDA values were reached with the diets containing GK at rates from 20 to 40 g/kg. In conclusion, dietary incorporation of 40 g/kg of GS and GK added separately or combined increased the plasma and meat α-tocopherol content. Furthermore, the combinations of GS and GK with a proportion of GK of at least 50% optimised α-tocopherol concentration both in plasma and in thigh meat and mitigated lipid oxidation in 7-day stored meat.

## 1. Introduction

More than a billion of tonnes of agricultural waste are generated annually in the world [1]. This waste represents a major concern since its accumulation can become an environmental nuisance. In order to reduce the pollution load and in the frame of the circular economy approach, the European Union has urged its member countries to increase agricultural byproducts recycling and valorization, for instance through the inclusion of these low-cost raw materials in livestock feed [2,3,4]. In particular, the winemaking process gives rise to a significant amount of waste (grape seeds, skins and stems). It is estimated that around 20% of the total weight of grapes used for winemaking results in grape pomace, which is mostly destined to feed animals or used as fertiliser [5]. Nevertheless, non-negligible quantities of bioactive substances remain in these residual vegetable tissues [6]. Phenolic acids, several flavonoids, monomers (catechin, epicatechin and gallocatechin) and superior phenolic compounds (proanthocyanidins or condensed tannins) have been found to be among the main bioactive constituents in grape byproducts [7,8]. A literature search revealed that the number of publications including the keywords “antioxidant” and “grape pomace” has strongly increased over the last 10 years, emphasizing thus the growing interest in this topic. In monogastric animals, it has been demonstrated that the dietary inclusion of grape byproducts can affect intestinal barrier and microbial ecosystem [9,10]. Additionally, grape phenolic compounds have also shown to be bioavailable and effective in reaching target tissues, which, among other effects, has made possible increases in α- and γ-tocopherol contents in plasma and meat and a reduction of meat lipid oxidation in chickens fed grape byproducts [2,11]. 

The polyphenol composition of each part of the grape pomace is different and may vary depending on the growing conditions, the climate, the maturity and the fermentation time [12,13]. Grape seeds (GS) show a greater concentration of total extractable polyphenols (TEP) than grape skins (GK) [14]. Moreover, GS and GK differ not only in the amount of phenolic compounds contained but also in the nature of these compounds. Specifically, GS contain higher amounts of flavanols (monomeric catechins and epicatechins, as well as oligomeric procyanidins), whereas GK present a larger variety of phenolic compounds, such as phenolic acids (e.g., caftaric acid, fertaric acid and coutaric acid) and anthocyanins [15]. Moreover, procyanidins present in GK show a degree of polymerization higher than that of the procyanidins contained in GS [16]. These differences in the nature of the phenolic compounds incorporated to the diet by GS or GK, as well the fermentation process of grape byproducts, affected differently the intestinal utilization of grape polyphenols and thereby their biological activity and antioxidant potential in chickens [11,13,17]. Furthermore, recent results [11] suggest that the dietary combination of a mixture of GS and GK, at 65% and 35%, respectively, in order to simulate the composition of grape pomace, enabled a better antioxidant potential than the separate inclusion of these grape byproducts. Indeed, synergistic, antagonistic and additive interactions among different kinds of polyphenol have been described [18,19]. Nevertheless, high dietary doses of grape polyphenols can also affect negatively nutrient digestibility and growth performance [13,20]. Therefore, it would be interesting to conduct further research to evaluate different combinations of GS and GK in an attempt to maximise the antioxidant potential of dietary grape polyphenols without impairing nutrient digestibility or chicken growth performance. 

Thus, the aim of this study was to evaluate the effect of combining different proportions of GS and GK in the diet of chickens on growth parameters, protein digestibility, TEP and condensed tannins contents in ileum and excreta, plasma and thigh meat α-tocopherol concentration and oxidative stability of meat in broiler chickens.

## 2. Materials and Methods

### 2.1. Grape Byproducts 

Hermanos Delgado organic winery (Socuéllamos, Ciudad Real, Spain) supplied the GS and GK meals from red grapes (*Vitis vinifera* var. Cencibel) used in this study. These grape byproducts were obtained from a vinification tank after 15 days of alcoholic fermentation and were dried in a rotary trommel with heated air at temperatures below 80 °C. Seeds and skins were mechanically separated (by sieving and air aspiration) and ground to particles as big as 1.5 mm. Before grinding, seeds were subjected to an expeller oil extraction process. The proximate composition and the characterization of phenolic compounds of GS and GK can be found in the previous study of Romero et al. [11]. Briefly, GS and GK contained, respectively, 19.6 and 16.3 g/100 g dry matter (DM) of crude protein (CP); 25.4 and 14.5 g/100 g DM of crude fibre; 5062 and 4500 cal/g of gross energy; 7.93 and 2.35 g of TEP (gallic acid equivalents/100 g DM) and 1.15 and 1.24 of condensed tannins (g cyanidin/100 g DM). Flavanols (from monomeric to tetrameric structures) and phenolics acids present in GS and GK were also reported in our previous study [11]. 

### 2.2. Birds and Diets 

A total of 150 male broiler Cobb chicks 1-day-old obtained from a commercial hatchery (Avícola Grau, Toledo, Spain) were housed in electrically-heated starter battery brooders in an environmentally-controlled room. Chicks were allocated to 30 cages, each cage containing five chicks, to receive six dietary treatments during 21 days with five replicates per treatment. Ingredient and nutrient compositions of diets are shown in Table 1. Diets were formulated to meet or exceed the minimum NRC [21] requirements for broiler chickens and to contain the same amount of energy, protein and fibre, being the latter achieved by incorporating straw into the formula at different doses. Celite (Celite Corp., Lompoc, CA, USA), a source of acid-insoluble ash (AIA), was added at 10 g/kg to all diets as an indigestible marker. Diets in mash form and water were provided *ad libitum* throughout the experiment. Experimental procedures were approved by the University Complutense of Madrid Animal Care and Ethics Committee (protocol code CEA-UCM 20/2012) in compliance with the guidelines for the Care and Use of Animals for Scientific Purposes of the Ministry of Agriculture, Fishery and Food. Experimental diets were as follows: (1) Control corn-soybean diet; (2) Diet including 20 g/kg of GS and 20 g/kg of GK; (3) Diet including 30 g/kg of GS and 10 g/kg of GK; (4) Diet including 10 g/kg of GS and 30 g/kg of GK; (5) GS diet including 40 g/kg of GS; (6) GK diet including 40 g/kg of GK. 

### 2.3. Collection of Samples and Measurements

Body weight and feed consumption were recorded by cage at the beginning and at the end of the experimental period, hence enabling the calculation of the feed conversion ratio. At 19 days of age, clean stainless steel collection trays were placed under each cage, and excreta from the birds were collected for 48 h. A subsample of excreta per cage was collected in polyethylene bags and freeze-dried (Telstar, Terrasa, Spain) for subsequent determination of total extractable polyphenol and tannin contents. At 21 days of age, seven birds were randomly selected from each treatment and blood samples, obtained by cardiac puncture, were collected in lithium heparin vacutainer tubes and centrifuged (2500× *g* for 15 min at 4 °C). The plasma obtained was stored at −80 °C for α-tocopherol determination. Thereafter, these 42 birds were slaughtered and their thigh meat was collected and used to determine the extent of lipid oxidation and α-tocopherol concentration. Tissue samples were individually sampled, wrapped in transparent oxygen-permeable polyvinyl chloride film (13,500 cm^3^/m^2^/day) and stored at −20 °C until required. Meat samples were thawed in a non-illuminated refrigerated cabinet at 4 °C and the progress of lipid oxidation and the α-tocopherol concentration were assessed in meat samples after 1 and 7 days of refrigerated storage. Another 15 birds per treatment (three birds per cage) were euthanized with carbon dioxide (100%), and the ileal content of the three birds of each replicate were collected, pooled and stored at −20 °C. Ileal contents were freeze-dried, ground (1 mm screen) and used to determine celite, protein, TEP and tannin contents. 

### 2.4. Chemical Analyses 

Dry matter (method 930.15), CP (method 968.06) and ether extract by Soxhlet fat analysis after acid hydrolysis (method 920.39) were analysed according to the procedures described for the Association of Official Analytical Chemists [22]. The AIA concentration in diet and ileal contents was measured after ashing the samples and treating the ashes with boiling 4 M HCl [23].

#### 2.4.1. Total Extractable Polyphenol and Tannin Contents

For the extraction of phenolic compounds in GS, GK, ileal digesta and excreta, 0.50 g of sample was suspended in 20 mL of acidic methanol/water (50:50 *v*/*v*, pH = 2), shaken at room temperature for 1 h and centrifuged (3500 rpm for 15 min at 4 °C). The supernatant was separated and the residue was resuspended in 20 mL of acetone/water (70:30 *v*/*v*), with shaking and centrifugation being repeated. The methanol and acetone extracts were combined and used to quantify TEP. The residue remaining after extraction was dried and used to determine tannin content. The TEP content was determined by the Folin–Ciocalteu procedure [24]. Briefly, 0.5 mL of extract, 0.5 mL of Folin–Ciocalteu reagent (Sigma-Aldrich, St. Louis, MO, USA) and 10 mL of 1 M Na_2_CO_3_ were mixed and after reacting for 1 h, absorbance was measured at 750 nm using an ultraviolet-visible spectrophotometer (Hitachi U-2000; Hitachi, Ltd., Tokyo, Japan). Absorbance values were compared against a standard curve made with gallic acid (Sigma-Aldrich, St. Louis, MO, USA) and results were expressed as grams of gallic acid equivalents (GAE) per 100 g of DM. 

The acidic butanol assay [25] was used to quantify the tannin content in the residues remained after the extraction of total extractable polyphenols in GS, GK, ileal digesta and excreta. Briefly, 50 mg of sample were mixed with 7 mL of a stock solution consisting of 0.07% (*w*/*v*) FeSO_4.7_ H_2_O dissolved in 95:5 (*v*/*v*) 1-butanol/HCl, heated at 95 °C for 50 min, cooled in an ice bath and centrifuged. Thereafter, the absorbance was measured at 550 nm and absorbance values were compared against a standard curve made with cyanidin-3-O-glucoside (Extrasynthese, Genay, France). Tannin content was expressed as cyanidin equivalents. 

#### 2.4.2. Plasma and Meat α-Tocopherol Quantification

The content of α-tocopherol in plasma and in meat stored under refrigeration for 1 and 7 days was determined following the method of Buttriss and Diplock [26]. Briefly, 400 µL of plasma or 100 mg of freeze-dried thigh meat were extracted with hexane in three phases, evaporated and diluted with 1 mL of hexane. The α-tocopherol content was measured by normal-phase HPLC using a Hypersil Si 100 (5 µm) column and a mobile phase of hexane-isopropanol (98:2 *v*/*v*) and detected by fluorescence using a HPLC system (Hewlett-Packard 1100, Agilent Technologies GmbH, Waldbronn, Germany).

#### 2.4.3. Meat Lipid Oxidation

The meat lipid oxidation was assessed by measuring the thiobarbituric acid reactive substances (TBARS) after 1 and 7 days of refrigerated storage of meat using the procedure described by Botsoglou et al. [27]. Five grams of ground meat were homogenized with 10 mL of 5% trichloroacetic acid containing 100 μL of 2% butylated hydroxytoluene in an Ultraturrax at 21,280× *g* for 1 min. The blended sample was centrifuged (3000 rpm for 5 min at 4 °C), filtered through Wathman number 2V filter (Whatman International Ltd., Maidstone, UK) and 2.5 mL of the filtrate were mixed with 1.5 mL of 0.8% thiobarbituric acid in capped test tubes, that were vortexed and incubated at 70 °C for 30 min. Absorbance was determined at 532 nm using an ultraviolet-visible spectrophotometer Hitachi U-2000 (Hitachi, Ltd., Chiyoda-ku, Tokyo). Results were expressed as mg of malondialdehyde (MDA) per kilogram of muscle after the preparation of a standard curve of 1,1,3,3-tetraethoxy propane.

### 2.5. Calculations and Statistical Analysis

Apparent ileal digestibility of CP was calculated using the following formula: 100% − [100% × (AIA concentration in feed/AIA concentration in ileal digesta) × (CP concentration in ileal digesta/CP concentration in feed)]. 

Data were subjected to a one-way analysis of variance (ANOVA) by using the general linear model procedure (Version 9.4, SAS Institute Inc., Cary, NC, USA). When the effect was declared significant (*p* < 0.05), means were compared using a Duncan’s multiple-range test.

## 3. Results

### 3.1. Growth Performance 

No significant differences among treatments were observed for daily weight gain, feed intake or feed conversion ratio (Table 2). The average values of these performance parameters were, respectively, 37.3 g/d, 55.5 g/d and 1.49 for broiler chickens from 1 to 21 days of age.

### 3.2. Intestinal Utilization of Protein and Polyphenols 

The digestibility results are shown in Table 3. Ileal digestibility of protein was not affected by dietary treatments, being on average 79.7%.

The ileal and excreta contents of TEP and condensed tannins are reported in Table 3. The dietary inclusion of grape byproducts increased (*p* < 0.001) TEP content both in the ileum and in the excreta as compared with the control birds. For birds fed the different grape byproducts diets, a similar average TEP concentration was found in the ileum (0.693 g GAE/100 g, on average). In the excreta, the average TEP content amounted to 0.626 g GAE/100 g for birds fed the grape byproducts diets. Among the different dietary treatments including grape byproducts, lower excreta TEP contents were obtained (*p* < 0.001) when birds were fed diets including GS at a dose from 20 g/kg. 

Likewise, the inclusion of grape byproducts in the diets also increased condensed tannin content in the ileum and in the excreta with respect to control birds (0.137 vs. 0.055 mg cyanidin/100 g in the ileum, *p* < 0.001, and 0.074 vs. 0.056 mg cyanidin/100 g in the excreta, *p* < 0.001). Both for ileal and for excreta tannin content, the highest dietary doses of GK (30 and 40 g/kg) resulted (*p* < 0.001) in the highest condensed tannin concentrations.

### 3.3. Plasma and Meat α-Tocopherol Concentration

Results for plasma and thigh meat α-tocopherol concentration are provided in Table 4. The dietary inclusion of grape byproducts increased plasma α-tocopherol concentration with respect to the control group (9.04 vs. 7.06 μg/mL, *p* < 0.001). Among the different grape byproduct treatments, the highest plasma α-tocopherol concentrations were reached with the 30 g/kg GS–10 g/kg GK and 20 g/kg GS–20 g/kg GK combinations. 

Similar to what was observed in plasma, both on day 1 and on day 7 of storage, α-tocopherol concentration was higher in the thigh meat of chickens fed the grape byproducts diets than in that of control birds (15.8 vs. 10.7 μg/g on day 1, *p* < 0.01, and 2.00 vs. 0.474 μg/g on day 7, *p* < 0.001). On day 1 of meat storage, no significant differences on meat α-tocopherol concentration were found among the grape byproducts treatments but, on day 7 of storage, the 20 g/kg GS–20 g/kg GK and 10 g/kg GS–30 g/kg GK combinations led (*p* < 0.001) to the highest values of α-tocopherol concentration in the thigh meat of chickens.

### 3.4. Meat Lipid Oxidation (Thiobarbituric Acid Reactive Substances, TBARS) 

The extent of meat lipid oxidation, measured as MDA, after 1 and 7 days of meat storage, is reported in Table 5. On day 1 of storage, no significant differences among the dietary treatments were detected for the MDA amount in broilers’ meat (0.159 mg MDA/kg, on average). After 7 days of storage, MDA values increased, almost by ten times, in chicken thigh meat (0.159 mg/kg on day 1 vs. 1.47 mg/kg on day 7, *p* < 0.001). On day 7 of storage, the MDA concentration was lower in the meat of chickens fed the grape byproducts diets than in that of control birds (1.27 vs. 2.49 mg MDA/kg, *p* < 0.001). Moreover, among the different grape byproducts treatments, the lowest MDA values were reached with the diets containing GK at rates from 20 to 40 g/kg.

## 4. Discussion

### 4.1. Growth Performance and Protein and Polyphenol Intestinal Utilization

In the present research work, the inclusion of 40 g/kg of GS and GK, individually or combined, in broiler chicken diets did not affect neither daily weight gain nor feed conversion ratio or ileal protein digestibility of birds. Accordingly, previous studies showed that feeding chickens with different grape byproducts (GK, [13]; GS, [11]; red and white grape pomace, [28,29]) up to 50 g/kg did not compromise chicken productive performance. However, higher doses of GK (60 and 110 g/kg, providing more than 0.74 g/kg of dietary tannins) reduced weight gain, impaired feed conversion ratio and decreased the ileal digestibility of protein in chickens [11,13]. In the present study, grape byproducts incorporated less than 0.5 g/kg of tannins in all treatments. Previous studies [11,13,30] also showed that the individual dietary inclusion of 30 g/kg of GS or GK, resulting in an incorporation of less than 0.40 g/kg of tannins to the diet, had no detrimental effect on growth performance nor on ileal protein digestibility of chickens. However, when GS and GK are combined in the diet (for instance, with the dietary inclusion of grape pomace), a higher dietary concentration of tannins can be reached before impairing chicken performance. Actually, the negative effects on chickens’ performance were only observed with a dietary tannins concentration from 2 g/kg (obtained with a dietary inclusion of 100 g/kg of grape pomace), whereas productive performance of chickens were not impaired if dietary tannin content remained around 1 g/kg (50 g/kg of grape pomace in the diet) [31]. Tannins have traditionally been deemed anti-nutritional factors in poultry [32], since they are known to bind to proteins due to the interaction of their reactive hydroxyl groups with the carbonyl groups of proteins, representing hence a hindrance for protein digestion and causing, consequently, an impairment of animal performance. The higher polymerization degree of the procyanidins present in grape skins might account for the negative effects observed, at a similar rate of tannins incorporated to the diet, with the dietary inclusion of grape skins but not with that of grape pomace. Grape byproducts present a variable content and nature of phenolic compounds, which makes it necessary to determine the level of dietary inclusion of grape byproducts that ensures no negative effect on growth performance. In this study, where different grape byproducts and various combinations were compared, we report that, regardless of their nature, an inclusion of 40 g/kg of grape byproducts seems to not compromise chicken growth performance. 

As expected, in the current experiment chickens fed the grape byproducts diets showed higher ileal and excreta TEP and tannin contents than birds fed the control diet. While no significant differences were detected for ileal TEP content among the different dietary treatments including grape byproducts, excreta TEP concentration was greater with high dietary doses of GK than with high doses of GS, which suggests that polyphenols from GK are utilised in the large intestine to a lesser extent than those originating from GS. Furthermore, dietary treatments including the highest dietary doses of GK (30 and 40 g/kg) also led to an increase in the tannin content in the ileum and in the excreta. Likewise, Romero et al. [11] found that the individual dietary inclusion of GK at 110 g/kg increased tannin concentration both in ileal and in excreta contents. Previous research [10] has demonstrated that undigested polyphenols reaching the large intestine can be utilized by means of microbial fermentation giving rise to small bioactive phenolic metabolites [33,34]. The different intestinal utilization detected for polyphenols of GS and GK could be due to the different phenolic composition of these polyphenol sources [35]. Specifically, GS contain higher amounts of monomeric and some oligomeric compounds, which are likely to be easily utilized in the gut, whereas GK include highly polymerized procyanidins that need to be hydrolysed by intestinal microbiota to produce more easily absorbable metabolites [36,37].

### 4.2. Plasma and Meat α-Tocopherol Concentration

The capacity of dietary grape polyphenols to increase α-tocopherol content in liver, plasma and meat of chickens had been reported in previous research [11,31,38]. Accordingly, in the present study, the inclusion of different grape byproducts in the diet of broiler chickens increased α-tocopherol concentration both in plasma and in stored meat with respect to control birds. 

Concerning plasma α-tocopherol concentration, the highest increases with respect to control animals were obtained when GS and GK were combined whereas no effect was observed when including only GS at 40 g/kg. The effect observed on α-tocopherol did not seem to be correlated with the total extractable grape polyphenol content provided by the different sources, since the different diets combining GS and GK incorporated to the diet grape polyphenols amounts (less than 2.6 g/kg) lower than that provided by the GS40 diet (3.2 g/kg). Likewise, in previous research works [11,31], increases in plasma α-tocopherol concentration were also obtained with diverse dietary grape polyphenols contents (from 1.17 to 2.27 g/kg), but in all these cases with grape polyphenol sources that included both GS and GK (by feeding either grape pomace or a combination of GS and GK). Furthermore, with grape byproduct diets presenting all the same dietary content of grape polyphenols, Romero et al. [11] also obtained a higher plasma α-tocopherol concentration in chickens if GK and GS were combined in the diet instead of being fed separately. On balance, it can be deduced that the increase of plasma α-tocopherol concentration cannot be attributed to the dietary amount of grape polyphenols, but rather to the kind of polyphenols present in the diet, since GK and GS provide different types of phenolic compound. The combined inclusion in the diet of both GS and GK would, hence, contribute to enriching the variety of phenolic compounds ingested by chickens. Synergistic and additive interactions among polyphenols have previously been described with other sources of flavan-3-ols, such as tea [18,19]. 

On the other hand, the increase of plasma α-tocopherol concentration might originate from the content itself of this vitamin in grape byproducts. Wie et al. [39] reported that the total concentration of α-tocopherol in grape seeds from 14 cultivars ranged from 4.8 to 9.9 mg/100 g. However, in the present study, we used a defatted grape seed meal and consequently, the amount of α-tocopherol that could have been provided by this grape byproduct would be negligible as compared with the total dietary vitamin E concentration. 

Alpha-tocopherol is the major antioxidant localized in muscle lipids and its level is crucial for the resistance of lipids to oxidation. In this experiment, the increase of α-tocopherol in plasma was correlated with an increase of α-tocopherol concentration in meat, with this effect being maintained after 7 days of refrigerated storage of meat. On day 1 of storage, the increase in meat α-tocopherol concentration with respect to the control group was similar among the different treatments containing grape byproducts. After 7 days of storage, as expected, meat α-tocopherol concentration decreased for all treatments but nonetheless, this reduction was less pronounced in the meat of birds fed grape byproducts, especially in those fed the diets combining GS and GK. In this sense, after 7 days of meat storage, the highest meat α-tocopherol concentrations were achieved with the combinations containing the higher proportions of GK (20 g/kg GS-20 g/kg GK and 10 g/kg GS-30 g/kg GK). Actually, this study confirms our previous findings [11] indicating that feeding a combination of GS and GK led to higher α-tocopherol concentrations than diets including separately GS or GK and also to a higher stability of the α-tocopherol present in meat.

### 4.3. Meat Lipid Oxidation

Regarding meat lipid oxidation, as expected, thigh meat MDA values increased, almost by 10 times, after 7 days of meat storage, with results being affected by dietary treatments. In this sense, on day 7 of storage, MDA values were lower in chickens fed the grape byproduct diets than in control birds. These effects were correlated with the greater content and stability of α-tocopherol observed in the meat of birds fed the grape byproducts diets. Additionally, in a parallel way to what has been observed for meat α-tocopherol content, the diets containing GK at doses ranging from 20 to 40 g/kg, which were actually the diets showing the lowest grape polyphenols concentrations (from 0.94 to 2.06 g/kg), resulted in the lowest MDA values in 7-day stored meat. These results suggest that, as occurring with α-tocopherol content, the effect of grape polyphenols on meat oxidative stability is more correlated with the source than with the level of the grape polyphenols included in the diet. 

Likewise, previous studies [13,28,38] also reduced MDA concentration in chicken stored meat with dietary doses of grape polyphenols ranging from 0.729 to 2.92 g/kg. Actually, in another study [11], three grape byproducts diets with similar grape polyphenol concentration (2.4 g/kg) but different grape polyphenol sources had different effects on chicken stored meat MDA concentration, since the dietary combination of GS and GK led to a MDA value lower than those achieved with the diets including solely GS. Therefore, as previously suggested for α-tocopherol concentrations, it could be surmised that better results in the prevention of chicken meat lipid oxidation are attained with dietary combinations of GS and GK in which GK account for, at least, 50% of total proportion. 

In general, GK contain proanthocyanidins with a degree of polymerization higher than that of the proanthocyanidins present in GS [40]. A positive correlation among the degree of polymerization of proanthocyanidins and their antioxidant potential has been reported [41,42]. Procyanidins may not require their intestinal absorption to be bioactive. In the present study, a higher content of tannins was also observed in the intestine of chickens fed diets containing GK. Gut bacteria, which are able to metabolise polymeric polyphenols [10], may produce in situ new phenolic compounds of smaller size likely to be more easily absorbed at the intestine and able to reach animal tissues [43,44].

On the other hand, the dietary combinations of GS and GK might enable gathering in the diet the various types of phenolic compound contained in these grape byproducts. This enlarged variety of phenolic compounds, which could be considered as prebiotics, allows the proliferation in the intestine of a more diverse bacterial population, since it has been demonstrated that grape polyphenols can be extensively utilized by the microbiota present in chicken gut [9,10]. As result of these microbial fermentations of the dietary grape polyphenols, a larger number and variety of bioactive metabolites may be generated [34]. These phenolic metabolites could exert an antioxidant effect either by sparing liposoluble vitamin E [45] or by reducing radicals (α-tocopheroxyl) and, thereby, recycling the oxidized α-tocopherol [46].

## 5. Conclusions

Dietary incorporation of 40 g/kg of GS and GK added separately or combined increased the plasma and meat α-tocopherol content. Furthermore, the combinations of GS and GK where the proportion of GK represented at least 50% optimised the α-tocopherol concentration and mitigated lipid oxidation in 7-day stored meat. The beneficial effects of grape byproducts on α-tocopherol content and meat oxidative stability were more correlated with the source than with the level of the total extractable grape polyphenols included in the diet.

## Figures and Tables

**Table 1:** Ingredients and nutrient composition of experimental diets (g/kg as fed).

Ingredients	Experimental Diets					
	Control	Control GS ^1^ 20 + GK ^2^ 20	Control GS30 + GK10	Control GS10 + GK30	Control + GS40	Control + GK40
Corn (8.1% crude protein)	451	459	463	454	468	453
Soybean (48% crude protein)	373	364	362	364	360	362
Sunflower oil	86.0	83.0	82.0	84.0	81.0	85.0
Salt	3.0	3.0	3.0	3.0	3.0	3.0
Monocalcium phosphate	15.3	15.2	15.5	15.5	15.5	15.5
Calcium carbonate	15.6	15.3	15.3	15.3	15.3	15.3
Vitamin-mineral premix ^3^	5.0	5.0	5.0	5.0	5.0	5.0
DL-Methionine	2.0	2.0	2.0	2.0	2.0	2.0
Straw	42.0	6.0	5.0	10.0	3.0	12.0
Grape seed	-	20.0	30.0	10.0	40.0	-
Grape skin	-	20.0	10.0	30.0	-	40.0
Celite ^4^	10.0	10.0	10.0	10.0	10.0	10.0
*Analysed composition*						
Crude protein	203	208	207	194	209	205
Ether extract	109	109	108	109	108	110
*Calculated composition*						
AME ^5^ (kcal/kg)	3095	3089	3089	3080	3093	3076
Crude fibre	46.0	46.0	46.0	46.0	46.0	46.0
Lysine	12.1	12.0	12.0	12.1	12.2	12.2
Meth+Cys	9.0	9.0	9.0	9.0	9.0	9.0
Calcium	10.0	10.0	10.0	10.0	11.0	11.0
Available P	4.5	4.5	4.5	4.5	4.5	4.5
Extractable grape polyphenols (g GAE ^6^/kg)	-	2.1	2.6	1.5	3.2	0.9
Condensed tannins (g cyanidin/kg)	-	0.48	0.47	0.49	0.46	0.50

^1^ GS = Grape seeds ^2^ GK = Grape skins ^3^ Vitamin-mineral mix supplied the following per kilogram of diet: vitamin A, 8250 IU; cholecalciferol, 1000 IU; vitamin E, 11 IU; vitamin K, 1.1 mg; vitamin B12, 12.5 μg; riboflavin, 5.5 mg; Ca panthothenate, 11 mg; niacin, 53.3 mg; choline chloride, 1020 mg; folic acid, 0.75 mg; biotin, 05 mg; delquin, 125 mg; DL-Met, 500 mg; amprol, 1 g; Mn, 55 mg; Zn, 50 mg; Fe, 80 mg; Cu, 5 mg; Se, 0.1 mg; I, 0.18 mg; and NaCl, 2500 mg ^4^ Celite Corp, Lompoc, CA ^5^ AME = apparent metabolisable energy ^6^ GAE = gallic acid equivalents.

**Table 2:** Effect of grape seed (GS) and grape skin (GK) meals added at different dietary concentrations (g/kg) on growth performance of broiler chickens (1 to 21 d).

Dietary Treatments	Daily Weight Gain (g/d)	Daily Feed Intake (g/d)	Feed Conversion Ratio
Control	37.9	55.6	1.46
GS20 + GK20	37.0	56.2	1.52
GS30 + GK10	36.8	52.8	1.44
GS10 + GK30	36.6	57.2	1.54
GS40	38.6	56.1	1.46
GK40	36.9	55.2	1.50
SEM ^1^	0.933	1.73	0.042
*p*-value ^2^	ns	ns	ns

^1^ SEM, standard error of the mean; each value represents the mean of five replicates (five birds per replicate). ^2^ ns: no significant effect (*p* > 0.05).

**Table 3:** Effect of grape seed (GS) and grape skin (GK) meals added at different dietary concentrations (g/kg) on ileal protein digestibility and on ileal and excreta total extractable polyphenols and condensed tannins contents in broiler chickens.

Dietary Treatments	Ileal Protein Digestibility (%)	Total Extractable Polyphenols (g GAE ^1^/100 g)		Condensed Tannins (mg Cyanidin/100 g)	
		Ileal	Excreta	Ileal	Excreta
Control	81.3	0.504 ^b^	0.462 ^c^	0.055 ^e^	0.056 ^c^
GS20 + GK20	78.5	0.680 ^a^	0.594 ^b^	0.140 ^bc^	0.065 ^b^
GS30 + GK10	77.7	0.675 ^a^	0.602 ^b^	0.121 ^cd^	0.069 ^b^
GS10 + GK30	78.6	0.686 ^a^	0.672 ^a^	0.164 ^ab^	0.083 ^a^
GS40	80.4	0.736 ^a^	0.591 ^b^	0.100 ^d^	0.066 ^b^
GK40	81.5	0.689 ^a^	0.669 ^a^	0.160 ^ab^	0.089 ^a^
SEM ^2^	1.70	0.024	0.019	0.009	0.003
*p*-value ^3^	ns	***	***	***	***

Different letters in the same column (a, b, c, d, e) indicate significant differences (*p* < 0.05). ^1^ GAE: gallic acid equivalents ^2^ SEM, standard error of the mean; each value represents the mean of five replicates (three birds per replicate for ileum and five birds per replicate for excreta). ^3^ ns: no significant effect (*p* > 0.05). *** *p* < 0.001.

**Table 4:** Effect of grape seed (GS) and grape skin (GK) meals added at different dietary concentrations (g/kg) on plasma and thigh meat α-tocopherol concentration in broiler chickens.

Dietary Treatments	Plasma α-Tocopherol (μg/mL)	Thigh Meat α-Tocopherol (μg/g)	
		1 d ^1^	7 d
Control	7.06 ^d^	10.7 ^b^	0.474 ^e^
GS20 + GK20	9.69 ^ab^	15.7 ^a^	2.92 ^a^
GS30 + GK10	10.3 ^a^	14.8 ^a^	1.28 ^d^
GS10 + GK30	8.55 ^bc^	14.8 ^a^	2.34 ^ab^
GS40	7.88 ^cd^	16.6 ^a^	1.38 ^cd^
GK40	8.80 ^bc^	17.2 ^a^	2.06 ^bc^
SEM ^2^	0.424	1.20	0.261
*p*-value ^3^	***	**	***

Different letters in the same column (a, b, c, d, e) indicate significant differences (*p* < 0.05). ^1^ One or seven days of meat storage ^2^ SEM, standard error of the mean; each value represents the mean of seven birds from each treatment for plasma α-tocopherol concentration. ^3^ ** *p* < 0.01; *** *p* < 0.001.

**Table 5:** Effect of grape seed (GS) and grape skin (GK) meals added at different dietary concentrations (g/kg) on stored thigh meat lipid oxidation in broiler chickens.

Dietary Treatments	TBARS (mg MDA/kg Meat)	
	1 d ^1^	7 d
Control	0.165	2.49 ^a^
GS20 + GK20	0.142	0.842 ^d^
GS30 + GK10	0.194	2.07 ^b^
GS10 + GK30	0.141	1.03 ^d^
GS40	0.170	1.62 ^c^
GK40	0.143	0.793 ^d^
SEM ^2^	0.016	0.145
*p*-value ^3^	ns	***

Different letters in the same column (a, b, c, d) indicate significant differences (*p* < 0.05). ^1^ One or seven days of meat storage ^2^ SEM, standard error of the mean; each value represents the mean of seven birds from each treatment for thigh α-tocopherol concentrations and thiobarbituric acid reactive substances (TBARS) determination. ^3^ ns: no significant effect (*p* > 0.05); *** *p* < 0.001.
